# Editorial: Recent advances in the management of cardiac disease in companion animals

**DOI:** 10.3389/fvets.2026.1881858

**Published:** 2026-06-05

**Authors:** Tomohiko Yoshida, Seijirow Goya, Yohei Mochizuki, Ryohei Suzuki

**Affiliations:** 1 Department of Small Animal Medical Center, Obihiro University of Agriculture and Veterinary Medicine, Obihiro, Japan; 2 Department of Bioresource Sciences, Nihon University, Fujisawa, Japan; 3 Faculty of Veterinary Medicine, Okayama University of Science, Imabari, Japan; 4 Laboratory of Veterinary Internal Medicine, School of Veterinary Science, Faculty of Veterinary Medicine, Nippon Veterinary and Life Science University, Musashino, Tokyo, Japan

**Keywords:** biomarkers, cardiology, pathophysiology, telemedicine, ultrasonography

## 1 Introduction

The paradigm of veterinary medicine has undergone a radical transformation over the last decade, shifting from reactive symptom management to a proactive, precision-based approach. This evolution is particularly evident in the field of cardiology and systemic internal medicine, where the integration of advanced imaging, novel biomarkers, and innovative pharmacology has redefined our diagnostic and therapeutic boundaries. The contemporary veterinary clinician is no longer limited to basic thoracic radiography or standard echocardiography; instead, they have access to a sophisticated toolkit that includes molecular diagnostics, real-time hemodynamic monitoring, and digital health technologies.

This Research Topic, titled “*Advanced diagnostic and therapeutic insights into animal cardiovascular and systemic disorders*,” was curated to reflect this multidisciplinary shift. It brings together 13 pioneering contributions that bridge the gap between benchtop pathophysiology and bedside clinical practice. The objective of this Research Topic is to emphasize the clinical utility of emerging technologies. These articles address critical challenges in feline and canine cardiology, as well as comparative medicine in species such as the Shetland pony, providing a comprehensive overview of the current state of the art. The diversity of these contributions—ranging from experimental models and large-scale prospective studies to meticulous case reports—underscores the necessity of an integrative approach to manage the complex interplay between cardiovascular health and systemic stability.

## 2 Optimization of management in acute and chronic heart failure

The cornerstone of heart failure management has traditionally relied on the “triple therapy” or “quadruple therapy” involving diuretics, ACE inhibitors, pimobendan, and spironolactone. However, the emergence of refractory cases and the increasing prevalence of cardio-renal syndrome have highlighted the limitations of conventional protocols. This section explores two groundbreaking approaches to optimizing heart failure management: the introduction of SGLT2 inhibitors and the application of point-of-care (POC) biomarker monitoring.

### 2.1 The emerging role of SGLT2 inhibitors in feline medicine

One of the most significant pharmacological advancements in human cardiology in recent years has been the discovery of the cardiovascular benefits of sodium-glucose cotransporter 2 (SGLT2) inhibitors. Initially developed as antidiabetic agents, drugs like dapagliflozin and bexagliflozin have demonstrated remarkable efficacy in reducing heart failure hospitalizations and cardiovascular mortality in human patients, regardless of their diabetic status. Kim and Song provide a pioneering contribution to feline medicine by describing the use of bexagliflozin in a cat suffering from both congestive heart failure (CHF) and advanced chronic kidney disease (CKD).

In cats, managing CHF in the presence of CKD is exceptionally challenging due to the delicate balance required for fluid removal. Excessive use of traditional loop diuretics like furosemide can exacerbate renal azotemia, leading to a therapeutic impasse. The study by Kim and Song highlights how bexagliflozin acted as an “adjunct decongestive strategy.” By promoting osmotic diuresis through glycosuria, SGLT2 inhibition provides a mechanism for fluid removal that is independent of the renin-angiotensin-aldosterone system (RAAS) activation typically seen with loop diuretics. Furthermore, the potential “fuel-switching” effect—where the heart utilizes ketones more efficiently—may provide additional myocardial support in the feline patient. This case serves as a crucial proof-of-concept, suggesting that SGLT2 inhibitors could be a game-changer for feline cardio-renal syndrome, warranting further large-scale clinical trials to establish definitive safety and efficacy profiles in the feline species.

### 2.2 POC lactate monitoring: a new window into hemodynamic stability

Effective heart failure management requires not only the right drugs but also the right monitoring tools. While echocardiography remains the gold standard for structural assessment, it often fails to provide a real-time snapshot of systemic tissue perfusion. Choi et al. addressed this gap by investigating the clinical utility of point-of-care (POC) lactate measurement in dogs with myxomatous mitral valve disease (MMVD).

Hyperlactatemia has long been recognized as a marker of anaerobic metabolism and poor prognosis in emergency settings. However, Choi et al. expanded this application to the longitudinal management of MMVD patients. Their research demonstrates that serial lactate measurements provide objective data that correlate with the severity of heart failure (ACVIM stages) and, more importantly, with the patient's immediate response to therapy. In dogs presented with acute decompensation, the “lactate clearance rate” served as a superior predictor of short-term survival compared to single-point measurements. By integrating this low-cost, rapid diagnostic tool into routine practice, clinicians can more accurately calibrate their decongestive therapy and provide pet owners with data-driven prognostic expectations. This study reinforces the importance of monitoring the “systemic footprint” of cardiac disease, ensuring that therapeutic interventions successfully translate into improved peripheral oxygen delivery.

## 3 Redefining the landscape of canine myxomatous mitral valve disease (MMVD)

Myxomatous mitral valve disease (MMVD) remains the most prevalent cardiac pathology in dogs worldwide, yet the scientific community continues to uncover new layers of its complexity. Beyond the primary valvular degeneration, the clinical progression of MMVD is dictated by compensatory remodeling, electrical instability, and systemic metabolic shifts.

### 3.1 Risk stratification in decompensated MMVD

The transition from Stage B2 to Stage C (congestive heart failure) marks a critical point in the patient's life. Yin et al. provide a comprehensive retrospective analysis of dogs hospitalized for cardiogenic pulmonary edema (CPE) due to MMVD. Their work identifies specific risk factors for clinical outcomes, including renal parameters and markers of severe left atrial enlargement. This study is particularly valuable because it moves beyond simple classification; it provides clinicians with a prognostic roadmap. By recognizing that certain clinical findings upon admission correlate with higher rehospitalization rates, veterinarians can implement more aggressive monitoring and tailor communication with owners regarding the long-term management of CPE.

### 3.2 Electrical instability: atrial fibrillation and beyond

Electrical remodeling is a hallmark of advanced MMVD, often manifesting as atrial fibrillation (AF) due to severe atrial stretch. However, the presence of concurrent ventricular arrhythmias often complicates the clinical picture. Romito et al. explored this phenomenon by comparing dogs with secondary AF with and without concomitant frequent and complex ventricular arrhythmias. Their findings suggest that dogs with ventricular arrhythmias exhibit more pronounced echocardiographic abnormalities, indicating a higher degree of myocardial insult. This research emphasizes that AF should not be viewed as an isolated electrical event; instead, it is a marker of profound structural and electrical failure that requires a comprehensive anti-arrhythmic and hemodynamic approach.

### 3.3 The heart-systemic interaction: the iron deficiency connection

Perhaps one of the most intriguing contributions to this Research Topic is the work of Kitazawa et al. regarding iron deficiency in MMVD. In human medicine, iron deficiency is a recognized prognostic factor in heart failure, regardless of the presence of anemia. Kitazawa et al. demonstrated a similar trend in dogs, finding a high prevalence of iron deficiency across various MMVD stages. Crucially, iron deficiency was associated with diminished cardiac function, independent of other hemodynamic variables. This study shifts the therapeutic focus from purely “mechanical” support (improving forward flow and reducing congestion) to “metabolic” support. It suggests that correcting iron deficits could potentially improve myocardial energetics and quality of life in canine heart failure patients, a hypothesis that warrants future prospective intervention trials.

## 4 Technological innovation: from experimental models to wearable biometrics

The ability to detect cardiac dysfunction before it becomes clinically apparent is the “holy grail” of veterinary cardiology. This section details how advanced signal processing and wearable technology are bringing us closer to this goal.

### 4.1 Nonlinear HRV: a new frontier in subclinical detection

While traditional echocardiographic measurements like ejection fraction and fractional shortening are standard, they often remain within reference intervals during the early stages of myocardial dysfunction. Hasegawa et al. utilized a doxorubicin-induced cardiomyopathy model to investigate the utility of nonlinear heart rate variability (HRV). Unlike linear HRV analysis, which may be influenced by various external factors, nonlinear indices provide a more robust assessment of the autonomic nervous system's complexity. Their study found that nonlinear HRV could identify early myocardial changes before they were detectable by conventional echocardiography. This underscores the potential for HRV-based monitoring as a sensitive, non-invasive screening tool for patients at risk of developing cardiomyopathy, such as those undergoing chemotherapy.

### 4.2 The AI-COLLAR study: establishing new standards for home monitoring

The stress of the clinical environment—the “white coat effect”—has always been a barrier to accurate cardiorespiratory assessment. Chetboul et al. (the AI-COLLAR study) addressed this through a massive international prospective study of 703 dogs using wearable biometric devices. This research established robust reference intervals for resting heart rate (RHR) and resting respiratory rate (RRR) in the dog's natural environment. By collecting data over long periods, the study accounts for diurnal variations and individual breed differences, providing a baseline that is far more representative than a single snapshot taken in a clinic. This study is transformative; it provides the scientific foundation for using digital health tools to monitor chronic cardiac patients remotely, allowing for earlier detection of decompensation and more personalized dose adjustments of diuretics.

## 5 Deciphering the complexity of feline cardiomyopathies and vascular complications

Feline cardiology is often characterized by its phenotypic heterogeneity and the clinical challenge of occult progression. Unlike dogs, cats frequently mask their symptoms until they reach a state of critical decompensation, making the insights provided in this section vital for early intervention and accurate diagnosis.

### 5.1 Harmonizing clinical and imaging phenotypes in HCM

Hypertrophic cardiomyopathy (HCM) is the most common heart disease in cats, yet its clinical presentation ranges from asymptomatic murmurs to sudden death. de Sousa et al. have provided a monumental review that serves as a cornerstone for this Research Topic. They meticulously link clinical diagnostic assessments with imaging phenotypes, emphasizing that HCM is not a monolithic disease but a spectrum of myocardial remodeling. Their review highlights the importance of integrating traditional 2D echocardiography with advanced techniques such as tissue Doppler imaging and strain analysis to detect longitudinal dysfunction. By synthesizing current evidence, the authors provide a diagnostic algorithm that helps clinicians navigate the “gray zones” of feline myocardial thickening, such as distinguishing between physiological remodeling and early-stage HCM.

### 5.2 The molecular link to thromboembolism: TLR4 and platelets

Arterial thromboembolism (ATE) is perhaps the most feared complication of feline cardiac disease. Li et al. have contributed a high-impact original research article that delves into the molecular mechanisms of feline platelet activation. Their study identifies that functional Toll-like receptor 4 (TLR4) on feline platelets serves as a critical link between endotoxin sensing and “platelet priming.” This finding is revolutionary because it suggests that systemic inflammation or subclinical endotoxemia could significantly lower the threshold for clot formation in cats with pre-existing heart disease. This “double-hit” hypothesis—where structural heart disease provides the stasis and systemic inflammation provides the priming—opens new avenues for antithrombotic research, moving beyond simple platelet inhibition toward targeting inflammatory pathways.

### 5.3 Rare congenital and structural pathologies: from septal defects to subaortic stenosis

While HCM dominates feline cardiology, rare structural anomalies continue to challenge our diagnostic skills. Lee et al. presented a rare case of concurrent atrial and ventricular septal defects in a young Sphynx cat, a breed already predisposed to various cardiac issues. This case underscores the necessity of a meticulous echocardiographic workup to identify multiple shunts, which can significantly alter the hemodynamic management of the patient. Furthermore, Cerbu et al. described a newly identified form of fixed subaortic stenosis in a feline patient, an anomaly more commonly associated with canine cardiology. These reports are essential as they remind the global veterinary community that “rare” does not mean “non-existent,” and they enrich our understanding of the anatomical diversity of feline cardiac disease.

## 6 Comparative medicine and the multi-systemic paradigm

Modern veterinary medicine increasingly recognizes that the heart does not function in isolation. The final contributions to this topic highlight the intersection of cardiology with systemic disorders and breed-specific variations.

### 6.1 Connective tissue disorders and systemic alterations

Bilgiç et al. reported a fascinating and complex case of an elderly dog exhibiting a constellation of skeletal and cardiovascular alterations. These findings were compatible with a systemic connective tissue disorder, drawing parallels to Marfan syndrome or Ehlers-Danlos syndrome in humans. This case highlights the importance of the “comparative medicine” lens; it encourages clinicians to look beyond the immediate cardiac findings (such as aortic root dilation) and consider whether they are part of a broader genetic or systemic pathology. Such insights are crucial for developing a comprehensive long-term care plan that addresses both mobility and cardiovascular stability.

### 6.2 Establishing standards in equine cardiology: the Shetland pony

Diagnostic accuracy in cardiology is inherently dependent on robust reference data, which must often be breed-specific. Matos et al. addressed a significant gap in equine medicine by providing an integrative anatomical and ultrasonographic assessment of the heart in Shetland ponies. By combining post-mortem anatomical measurements with live 2D echocardiography, they established precise reference intervals tailored to this specific breed. This work ensures that clinicians can confidently distinguish between normal variations in small equines and true pathological changes, thereby preventing both underdiagnosis and unnecessary treatment.

## 7 Conclusion

The articles collected in this Research Topic illustrate the vital synergy between high-tech diagnostic tools and diligent clinical observation. By integrating molecular insights, advanced imaging, and long-term monitoring, these studies collectively advance our ability to diagnose earlier, treat more effectively, and understand the complex systemic interactions of cardiovascular disease in animals. We hope this Research Topic serves as a catalyst for future research in the pursuit of improving the quality of life for our veterinary patients.

